# Supplementary material for: Distinct amyloid fibril structures formed by ALS-causing SOD1 mutants G93A and D101N
Source: EMBO Rep. 2025 Aug 26;26(19):4820–46. doi: 10.1038/s44319-025-00557-8 (PMC12508129; doi:10.1038/s44319-025-00557-8)
Supplement: Supplementary file 1 — Appendix [file 44319_2025_557_MOESM1_ESM.pdf]

Appendix for

Distinct amyloid fibril structures formed by ALS-causing SOD1 mutants G93A and D101N

Mu-Ya Zhang, Yeyang Ma, Li-Qiang Wang*, Wencheng Xia, Xiang-Ning Li, Kun Zhao, Jie Chen, Dan Li, Liangyu Zou, Zhengzhi Wang, Cong Liu*, Yi Liang*

* Corresponding author: Li-Qiang Wang, wangliqiang@whu.edu.cn; Cong Liu, liulab@sioc.ac.cn; Yi Liang, liangyi@whu.edu.cn

List of Appendix Figures and Tables

Appendix Tables

Appendix Table S1 Page 2

Appendix Figures

Appendix Figure S1 Page 3

Appendix Figure S2 Page 4

Appendix Figure S3 Page 5

Appendix Table S1. The primers designed for full-length human SOD1 with G93A mutation or D101N mutation.

S-G93A	5' GCTGACAAAGATGCTGTGGCCG3'
A-G93A	5' ACAGCATCTTTGTCAGCAGTCAC3'
S-D101N	5' GTCTATTGAAAATTCTGTGATCTCACTCTC3'
A-D101N	5' ACAGAATTTTCAATAGACACATCGGCC3'

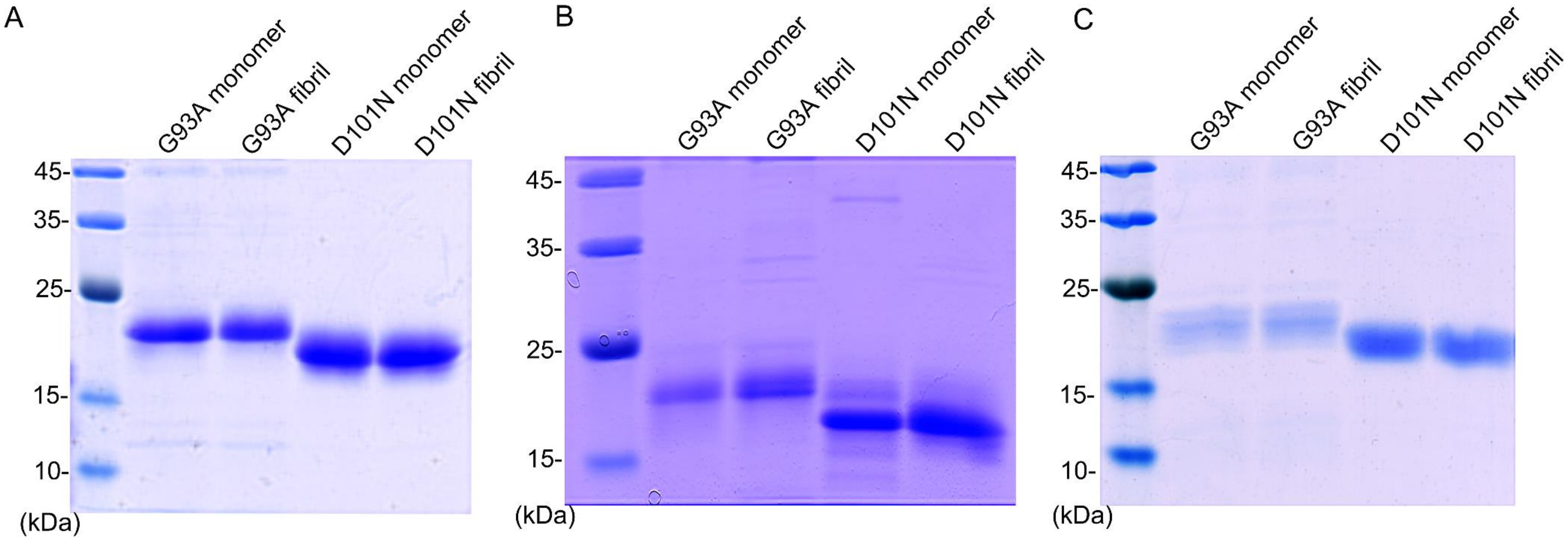

Appendix Figure S1. The SDS–PAGE gels of SOD1 protein before (G93A monomer and D101N monomer) and after *in vitro* aggregation (G93A fibril and D101N fibril) have been added to show the intact of the protein.

(**A**) SDS-PAGE analysis of SOD1 protein before and after *in vitro* aggregation have been added. In brief, the samples of G93A dimers, D101N dimers, G93A fibrils, and D101N fibrils were dissolved in 8 M urea and separated by 12.5% SDS–PAGE. The gels were stained with Coomassie Blue staining solution and washed with destaining buffer. All SDS–PAGE experiments were repeated three times and the results were reproducible. **B** and **C** represent two of the biological replicates of **A**. Marks at the left of the gels indicate the positions of the molecular weight markers. The SDS–PAGE experiments show that the protein was not degraded though no proteinase inhibitors were used during apo-SOD1 protein purification.

A

	Bacterial-purified SOD1			Expi293F cell–purified SOD1		
	G93A fibril	D101N fibril	Wild-type SOD1 fibril	G93A fibril	D101N fibril	Wild-type SOD1 fibril
$OD_{450\ nm}$	0.832±0.077	0.873±0.092	0.892±0.060	0.866±0.022	0.877±0.032	0.864±0.079
Concentration (mg/ml)	0.667±0.061	0.700±0.073	0.714±0.048	0.693±0.017	0.702±0.025	0.692±0.063

ELISA assay was used for accurately measuring the concentration of monomers denatured from the fibrils.

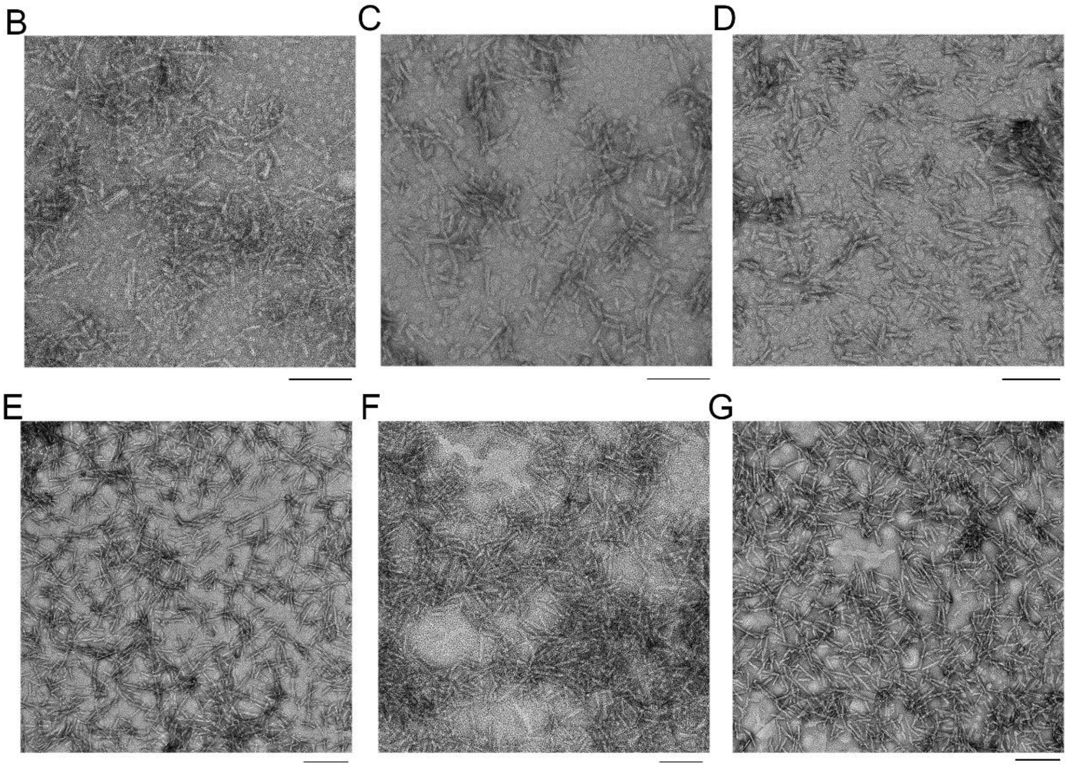

Appendix Figure S2. Accurate measurement of the concentration of monomers denatured from the SOD1 fibrils (A) and NS-EM images of the input fibrils for G93A, D101N and wild-type SOD1 fibrils (B–G).

(**A**) The samples of G93A fibrils, D101N fibrils, and wild-type SOD1 fibrils were dissolved in 8 M urea and then ELISA assay was used for accurately measuring the concentration of monomers denatured from the SOD1 fibrils. The concentration of fibril seeds from bacterial-purified SOD1 proteins and the mammalian cell-purified SOD1 proteins, measured by ELISA assay, was 0.667–0.714 mg/ml and 0.692–0.702 mg/ml, respectively, slightly smaller than that measured using NanoDrop (0.72 mg/ml). (**B**–**G**) Negative-stain electron microscopy (NS-EM) images of the input fibrils for G93A (**B** and **E**), D101N (**C** and **F**), and wild-type SOD1 (**D** and **G**) fibrils formed by bacterial-purified SOD1 proteins (**B**–**D**) and by the mammalian cell-purified SOD1 proteins (**E**–**G**). Abundant short fibrils with similar lengths were observed. Scale bars, 200 nm.

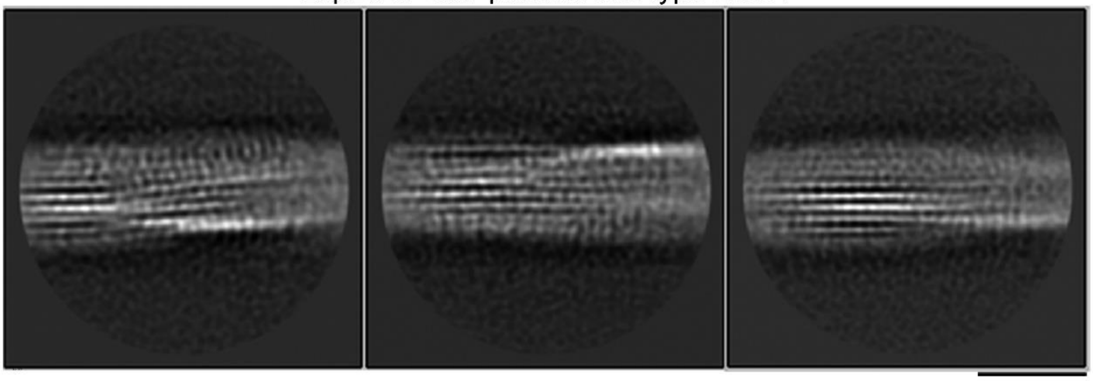

Appendix Figure S3. Cryo-EM images of wild-type SOD1 fibril.

Reference-free 2D class averages of the wild-type SOD1 fibril formed by Expi293F cell–purified wild-type SOD1 showing a single protofilament intertwined. Scale bar, 10 nm.
