# Supplementary material for: Distinct amyloid fibril structures formed by ALS-causing SOD1 mutants G93A and D101N
Source: EMBO Rep. 2025 Aug 26;26(19):4820–46. doi: 10.1038/s44319-025-00557-8 (PMC12508129; doi:10.1038/s44319-025-00557-8)
Supplement: Supplementary file 6 — Expanded View Figures [file 44319_2025_557_MOESM6_ESM.pdf]

Expanded View Figures

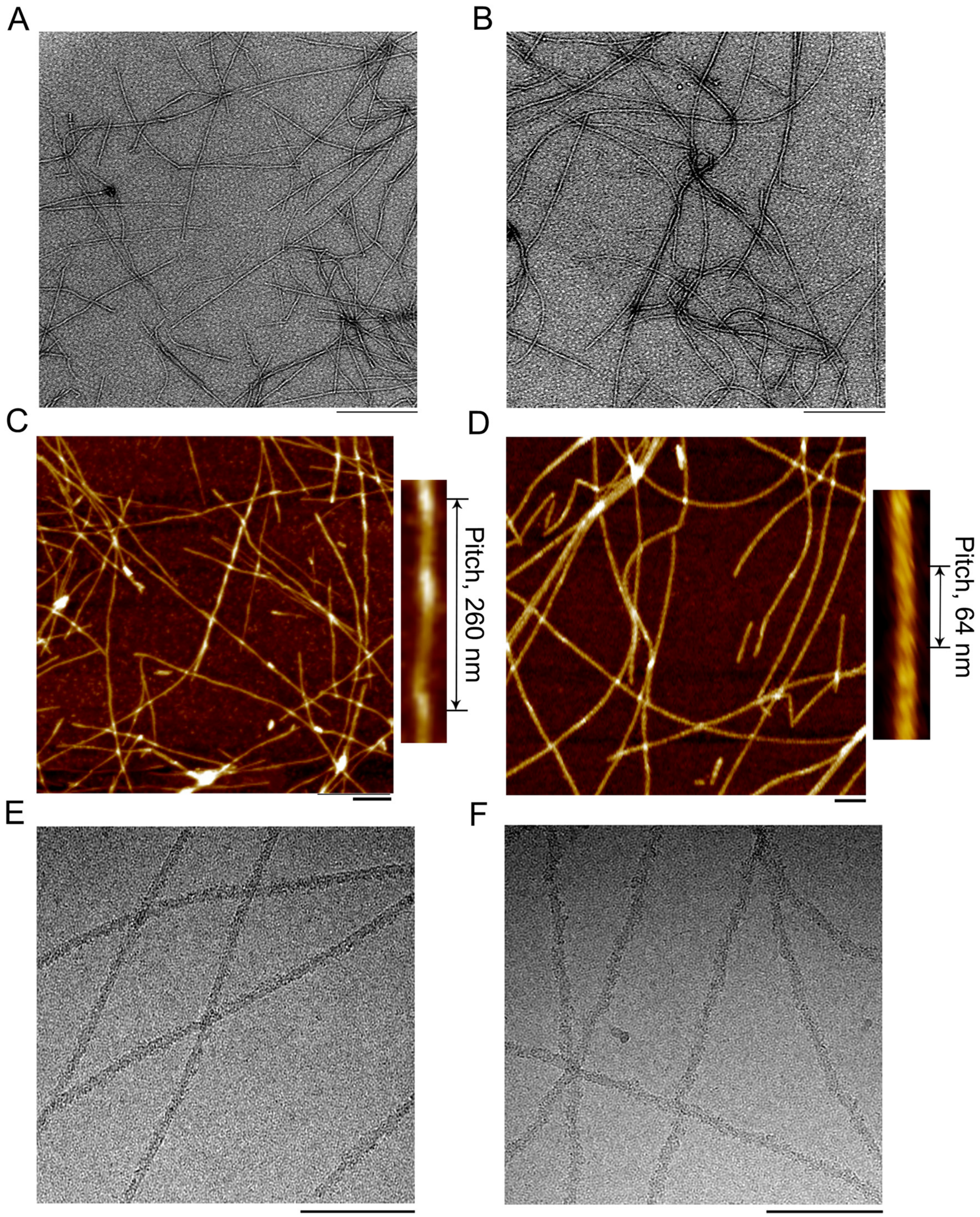

Figure EV1. Comparison of the images of G93A fibrils and D101N fibrils.

(**A**, **B**) Negative-staining TEM images of amyloid fibrils produced from the ALS-causing SOD1 mutant proteins G93A (**A**) and D101N (**B**). (**C**, **D**) AFM images of amyloid fibrils assembled from G93A (**C**) and D101N (**D**). The enlarged sections of (**C**) and (**D**) (right) showing the G93A fibril (**C**) and the D101N fibril (**D**) intertwined into a left-handed helix, with a helical pitch of 243 ± 11 nm and 64.3 ± 4.5 nm, respectively. The helical pitch was measured and expressed as the mean ± SD of values obtained in $n = 8$ biologically independent measurements. (**E**, **F**) Raw cryo-EM images of amyloid fibrils assembled from G93A (**E**) and D101N (**F**). The scale bars represent 200 nm (**A–D**) and 100 nm (**E**, **F**), respectively. Source data are available online for this figure.

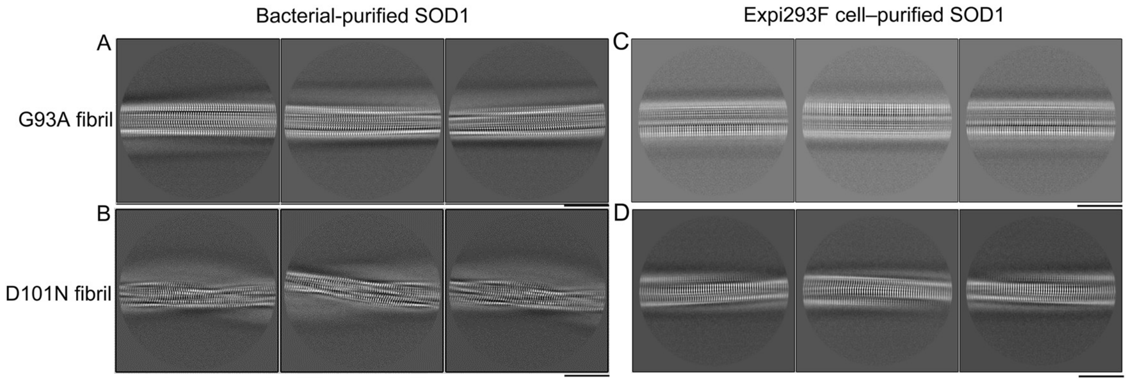

Figure EV2. Comparison of the cryo-EM images of G93A fibril and D101N fibril.

(**A**, **B**) Reference-free 2D class averages of the G93A fibril (**A**) and the D101N fibril (**B**) formed by bacterial-purified SOD1 mutants both showing a single protofilament intertwined. (**C**, **D**) Reference-free 2D class averages of the G93A fibril (**C**) and the D101N fibril (**D**) formed by Expi293F cell-purified SOD1 mutants showing a single protofilament less intertwined (**C**) and intertwined (**D**), respectively. Scale bars, 10 nm.

 © The Author(s)

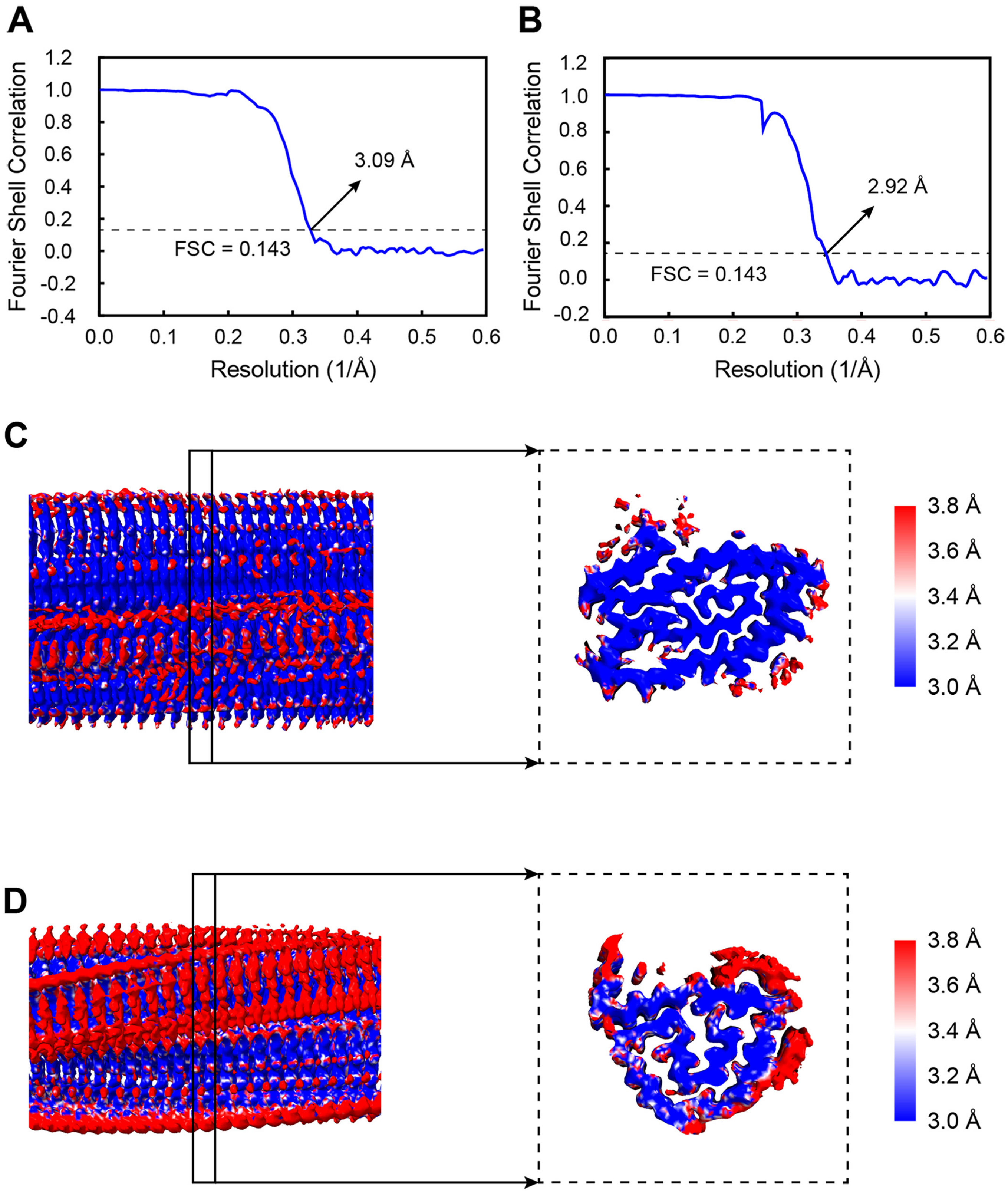
A
Fourier Shell Correlation
Resolution (1/Å)
3.09 Å
FSC = 0.143
B
Fourier Shell Correlation
Resolution (1/Å)
2.92 Å
FSC = 0.143
C
3.8 Å
3.6 Å
3.4 Å
3.2 Å
3.0 Å
D
3.8 Å
3.6 Å
3.4 Å
3.2 Å
3.0 Å

Figure EV3. Global (A and B) and local resolution (C and D) estimates for the reconstructions of G93A fibril and D101N fibril.

(**A**, **B**) The reconstruction was reworked and gold-standard refinement was used for estimation of the density map resolution. The global resolutions of 3.09 Å for the G93A fibril (**A**) and 2.92 Å for the D101N fibril (**B**) were calculated using two Fourier shell correlation (FSC) curves (blue) cut-off at 0.143. (**C**, **D**) The density maps of G93A fibrils (**C**) and D101N fibrils (**D**) are colored according to local resolution estimated by ResMap. The enlarged cross sections show the left top view of the density maps of a single protofilament in the G93A fibril (**C**) and a single protofilament in the D101N fibril (**D**). The color keys on the right show the local structural resolution in angstroms (Å) and the colored maps indicate the local resolution ranging from 3.0 to 3.8 Å.

© The Author(s)

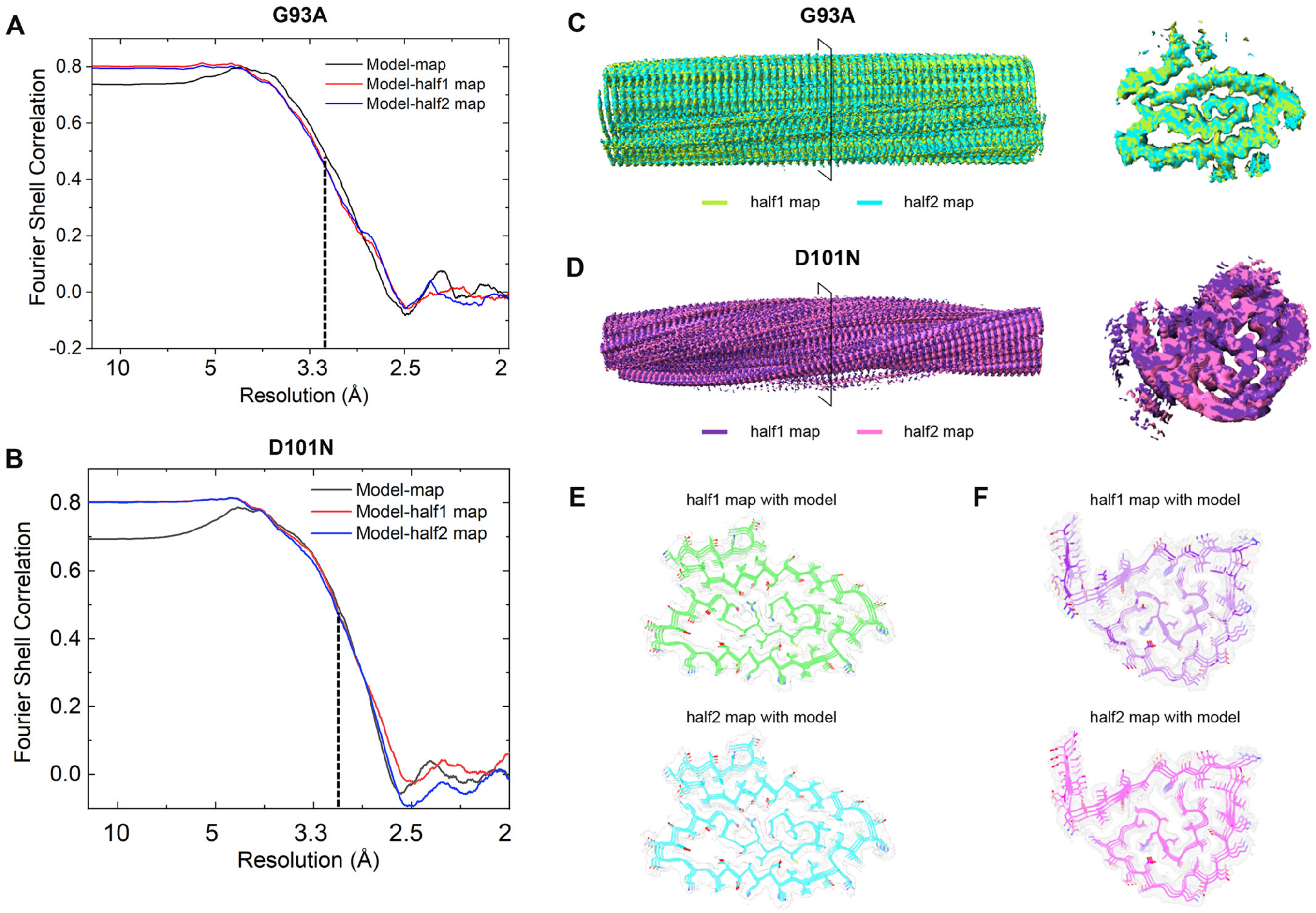

Figure EV4. For each structure of G93A fibril and D101N fibril, separate model refinements were performed against a single half-map, and the resulting model was compared with the other half-map to confirm the absence of overfitting.

(**A**, **B**) Fourier shell correlation (FSC) curves between the density map and the model. The FSC curves between the final refined model and the map reconstructed from all fibrils (black curve); between a model refined against the first half of the two independent half maps used for gold-standard FSC *versus* the reconstruction from that same half (red curve); and between a model refined against the first half of the two independent half maps *versus* the second independent half map (blue curve). The vertical lines at 3.09 Å (**A**) and 2.92 Å (**B**) indicate the highest resolution used in model refinement of the G93A fibril and the D101N fibril, respectively. These data convincingly demonstrate the absence of overfitting. (**C**, **D**) Comparison of the two optimized half-maps, half1 map (green and purple, respectively) and half2 map (cyan and magenta, respectively), from the G93A fibril (**C**) and the D101N fibril (**D**) 3D auto-refine process without mask, and the two half-maps match well. (**E**) Comparison of half1 map (green) and half2 map (cyan) of the G93A fibril with the atomic model overlaid. (**F**) Comparison of half1 map (purple) and half2 map (magenta) of the D101N fibril with the atomic model overlaid.

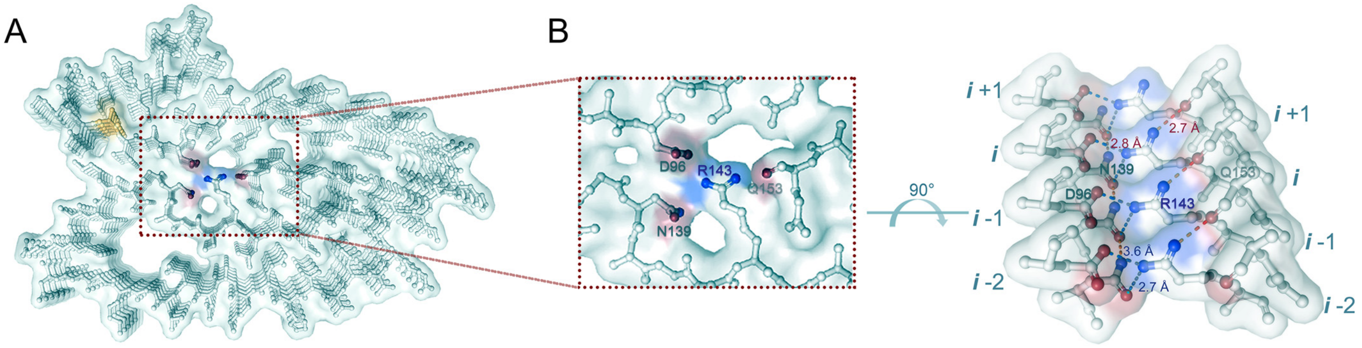

Figure EV5. Close-up view of the stick representation of the structure of G93A fibril stabilized by an intramolecular salt bridge and two hydrogen bonds.

(**A**) A space-filled model overlaid onto a stick representation of the G93A fibril in which a single protofilament containing five molecular layers is shown in light green. The ALS-causing mutation site Ala93 is highlighted in yellow. Asp96/Arg143 pairs that form a new salt bridge are highlighted in red (oxygen atom in Asp96) and blue (nitrogen atom in Arg143), and the salt bridge region is magnified in (**B**). Asn139/Arg143 pairs and Gln153/Arg143 pairs that form hydrogen bonds are highlighted in red (oxygen atoms in Asn139 and Gln153) and blue (two nitrogen atoms in Arg143), and two hydrogen bond regions are also magnified in (**B**). (**B**) A magnified top view of the salt bridge region of a G93A protofilament, where Asp96/Arg143 pairs form a salt bridge. A side view (right) highlighting a strong salt bridge between Arg143 and Asp96 from the same molecular layer, with a distance of 2.7 Å (blue). Magnified top views of the two hydrogen bond regions of a G93A protofilament, where two pairs of amino acids (Asn139 and Arg143, and Gln153 and Arg143) form two hydrogen bonds. Two side views (right) highlighting a hydrogen bond between Arg143 and Asn139 from the same molecular layer, with a distance of 3.6 Å (blue), or between Arg143 from the molecular layer (*i*) and Gln153 from the adjacent molecular layer (*i* - 1), with a distance of 2.7 Å (red). The orientation of the structure of G93A fibril in (**A**, **B**) has been flipped 180° along X-axis so that it is the same as that in Fig. 2.

 © The Author(s)

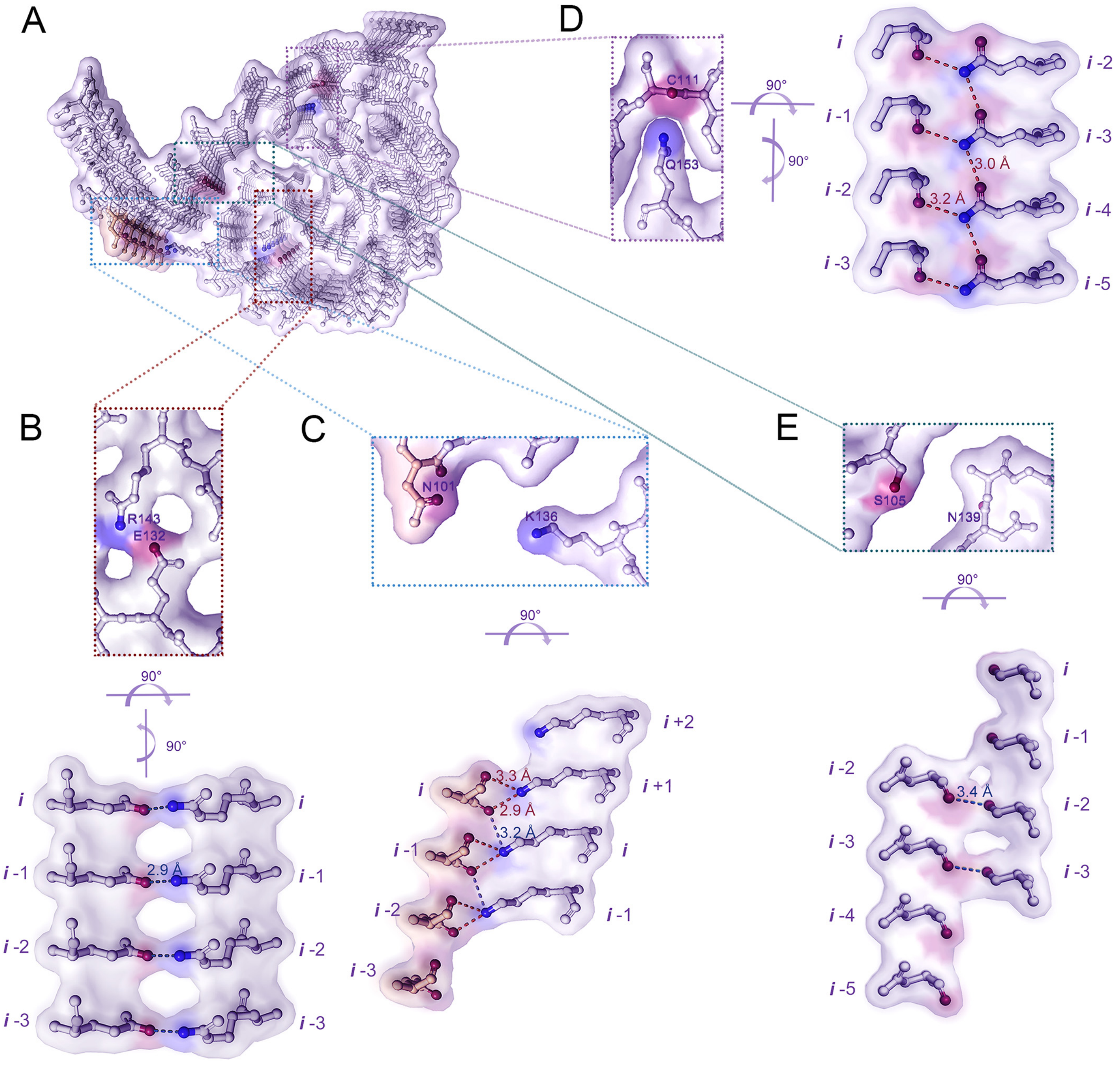

Figure EV6. Close-up view of the stick representation of the structure of D101N fibril stabilized by an intramolecular salt bridge and four hydrogen bonds.

(**A**) A space-filled model overlaid onto a stick representation of the D101N fibril in which a single protofilament is shown in light purple. The ALS-causing mutation site Asn101 is highlighted in gold. Asp132/Arg143 pairs that form a new salt bridge are highlighted in red (oxygen atom in Asp132) and blue (nitrogen atom in Arg143), and the salt bridge region is magnified in (**B**). Asn101/Lys136 pairs and Cys111/Gln153 pairs that form hydrogen bonds are highlighted in red (two oxygen atoms in Asn101 and oxygen atoms in Cys111) and blue (nitrogen atoms in Lys136 and Gln153), Asn139/Ser105 pairs that form a hydrogen bond are highlighted in red (oxygen atom in Asn139) and red (hydroxy group in Ser105), and three hydrogen bond regions are magnified in (**C**–**E**). (**B**) A magnified top view of the salt bridge region of a D101N protofilament, where Asp132/Arg143 pairs form a salt bridge. A side view (bottom) highlighting a strong salt bridge between Arg143 and Asp132 from the same molecular layer, with a distance of 2.9 Å (blue). (**C**) A magnified top view of a hydrogen bond region of a D101N protofilament, where Asn101/Lys136 pairs form two hydrogen bonds. A side view (bottom) highlighting a hydrogen bond between the main chain of Asn101 from the molecular layer (*i*) and Lys136 from the adjacent molecular layer ($i + 1$), with a distance of 2.9 Å (red), or between Asn101 from the molecular layer (*i*) and Lys136 from the adjacent molecular layer ($i + 1$), with a distance of 3.3 Å (red). (**D**) A magnified top view of a hydrogen bond region of a D101N protofilament, where Cys111/Gln153 pairs form a hydrogen bond. A side view (right) highlighting a hydrogen bond between the main chain of Cys111 from the molecular layer (*i*) and Gln153 from the molecular layer (*i* - 2), with a distance of 3.2 Å (red). (**E**) A magnified top view of a hydrogen bond region of a D101N protofilament, where Asn139/Ser105 pairs form a hydrogen bond. A side view (bottom) highlighting a hydrogen bond between the main chain of Asn139 and the hydroxy group in Ser105 from the same molecular layer, with a distance of 3.4 Å (blue).

© The Author(s)
